# Prophylactic use of a combination of palonosetron hydrochloride and dexamethasone to prevent transcatheter arterial chemoembolization-induced nausea and vomiting among patients with hepatocellular carcinoma

**DOI:** 10.3389/fmed.2025.1643731

**Published:** 2026-01-12

**Authors:** Wei-Mei Zhang, Meng-Jing Zheng, Cheng Li, Lei-Lei Chen, Chang Yu, Jiang-Fan Zhou, Jie Zheng

**Affiliations:** 1 Interventional Nursing Unit Interventional Surgery Center, The First Affiliated Hospital of Wenzhou Medical University, Wenzhou, China; 2 Interventional Nursing Unit 322 Ward, The First Affiliated Hospital of Wenzhou Medical University, Wenzhou, China; 3 Department of Intervention, The First Affiliated Hospital of Wenzhou Medical University, Wenzhou, China

**Keywords:** dexamethasone, transcatheter arterial chemoembolization, nausea, palonosetron hydrochloride, hepatocellular carcinoma, vomiting

## Abstract

**Objective:**

The effects of prophylactic administration of preclinical palonosetron hydrochloride (PH) and dexamethasone (DXMS) on nausea and vomiting (N/V) induced by transcatheter arterial chemoembolization (TACE) in patients with hepatocellular carcinoma (HCC) were evaluated. The goal was to furnish evidence that could guide clinical nursing practices.

**Methods:**

A retrospective analysis was conducted on 112 patients with HCC between July and December 2022. They were categorized into two groups: those administered PH only (the PH group) and those administered a combination of PH and DXMS (the PH + DXMS group). Statistical tests including *χ*^2^, Fisher’s exact test, paired *t*-test, and logistic regression were employed to compare the occurrence of N/V and identify associated factors.

**Results:**

After TACE was administered, there was a significant increase in the occurrence of nausea (*p* < 0.001) and vomiting (*p* < 0.003) among patients in the PH group. Compared with the PH group, the frequency of nausea after TACE among patients in the PH + DXMS group was significantly reduced (67.2% vs. 37.8%, respectively; *p* = 0.004), and the frequency of vomiting was slightly reduced (26.9% vs. 8.9%, respectively; *p* = 0.060). Univariate analysis revealed that the prophylactic use of PH + DXMS prior to interventional treatment significantly reduced the days of hospitalization in patients with HCC experiencing intervention-induced nausea (*p* = 0.031). Logistic regression analysis revealed that the level of alanine aminotransferase (ALT) before TACE (OR = 5.833, 95% CI: 1.252–27.170, *p* = 0.025) was an independent factor associated with the incidence of nausea.

**Conclusion:**

Prophylactic administration of a combination of PH and DXMS reduced the frequency of N/V as well as the length of hospital stay in patients with HCC. Moreover, the level of ALT before TACE was identified as a predictive factor for the incidence of nausea. Further research endeavors should explore patient-specific characteristics to optimize management strategies.

## 1 Introduction

Liver cancer stands as a prominent contributor to cancer-related mortality, ranking among the leading causes of cancer deaths in 46 countries and one of the top five causes in 90 countries worldwide (1). As per medical data for 2020, more than 900,000 people worldwide were diagnosed with liver cancer, resulting in over 830,000 deaths associated with the disease. China accounted for a substantial proportion, representing 45.3% of liver cancer cases worldwide and 47.1% of related deaths (2). Liver cancer is characterized by a high incidence rate, rapid progression, and pronounced invasiveness. Primary liver cancer (PLC) mainly comprises hepatocellular carcinoma (HCC), intrahepatic cholangiocarcinoma (ICC), and combined hepatocellular-cholangiocarcinoma (cHCC-CCA), with HCC representing 75–85% of all cases (3–5). Despite advances in understanding its etiology, which includes viral hepatitis, liver cirrhosis, and environmental factors, the prognosis of HCC remains poor due to late-stage diagnosis and high malignancy (6, 7).

Surgical intervention offers the best chance of long-term survival. However, many patients present with unresectable disease. Therefore, interventional treatments such as transcatheter arterial chemoembolization (TACE) have become standard for intermediate- to advanced-stage HCC. Kudo et al. (8) showed that the 1-year and 2-year overall survival of patients with HCC treated with TACE alone were 77.2 and 64.6%, respectively. Nevertheless, TACE is frequently accompanied by a series of adverse reactions, including nausea, vomiting, anorexia, fever, and other symptoms during the first 2 weeks of chemotherapy (9). Nausea and vomiting (N/V) caused by interventional therapy not only induce sensory discomfort for patients, but also lead to postoperative complications such as electrolyte imbalance, increased abdominal pressure, and malnutrition in severe cases.

Current international guidelines recommend prophylactic use of 5-hydroxytryptamine 3 receptor antagonists (5-HT3RA), dexamethasone (DXMS), and neurokinin-1 (NK1) receptor antagonists to prevent chemotherapy-induced N/V (10). The DXMS regimen was found to be more effective than the control regimen in preventing TACE-induced fever, anorexia, and N/V in patients with HCC (11). While DXMS is widely used for this purpose, the optimal antiemetic strategy specifically in the context of TACE for HCC patients has not been conclusively established. Palonosetron hydrochloride (PH) is a new type of highly selective and high-affinity second-generation 5-HT3RA antiemetic drug that can block presynaptic 5-HT in peripheral neurons of the emetic reflex center (12). It has emerged as a promising alternative due to its higher receptor binding affinity and longer half-life when compared with first-generation drugs (13). However, evidence regarding the combined prophylactic use of PH and DXMS in HCC patients undergoing TACE remains limited, and factors influencing patient responses are not well defined.

Therefore, this study was designed as a retrospective evaluation to explore the efficacy of prophylactic PH + DXMS in reducing N/V compared with PH alone in patients with HCC undergoing TACE, and to explore predictive factors to guide individualized antiemetic therapy.

## 2 Methods

This retrospective study analyzed clinical data from patients diagnosed with HCC who underwent their initial TACE treatment at the First Affiliated Hospital of Wenzhou Medical University between July 2022 and December 2022. Patients were stratified into two cohorts: the PH + DXMS group (*n* = 45) and the PH group (*n* = 67). The inclusion criteria included patients aged 18–85 years, diagnosed with HCC confirmed by imaging and histology, and met the criteria for TACE treatment. Exclusion criteria included a history of severe allergic reactions to steroids, presence of other malignancies, severe cardiopulmonary diseases or patients unable to tolerate TACE, and severe liver or renal dysfunction (Child-Pugh class C or severe hepatic encephalopathy). All participants received 0.5 mg of PH orally once daily, starting 1 day before the standard TACE procedure. In addition to PH, patients in the PH + DXMS group were administered 5 mg of DXMS intravenously 30 min prior to the intervention. Liver function was evaluated using the Child-Pugh classification, which examines five clinical parameters: general health status, the presence of ascites, serum bilirubin concentration, serum albumin levels, and prothrombin time. The tumor stage was categorized according to the Barcelona Clinic Liver Cancer (BCLC) staging system, incorporating tumor size, vascular invasion, lymph node involvement, distant metastasis, and liver function based on the Child-Pugh score.

This study received approval from the Ethics Committee of the First Affiliated Hospital of Wenzhou Medical University and was conducted in compliance with the ethical standards established by the *Helsinki Declaration*.

### 2.1 Definition and grading of N/V

Nausea was defined as an uncomfortable sensation often accompanied by an urge to vomit. It was categorized into four distinct severity grades: Grade 0 (no nausea), Grade 1 (reduced appetite but no significant change in food intake), Grade 2 (reduced oral intake without noticeable weight loss, dehydration, or malnutrition), and Grade 3 (severely insufficient oral intake necessitating nasogastric feeding, total parenteral nutrition, or hospitalization).

Vomiting was classified as the involuntary expulsion of stomach contents through the mouth. The severity was graded on a five-point scale: Grade 0 (no vomiting), Grade 1 (one to two vomiting episodes within a 24-h period, with at least 5 min between episodes), Grade 2 (three to five episodes within 24 h, maintaining intervals of at least 5 min), Grade 3 (six or more episodes within 24 h, with a minimum 5-min interval), and Grade 4 (life-threatening vomiting requiring immediate medical intervention).

Both nausea and vomiting were assessed through a combination of patient self-reports and clinical observations by healthcare professionals to minimize variability.

### 2.2 Statistical analysis

Data were compiled and analyzed using SPSS version 25.0. Descriptive statistics were applied to present general patient characteristics and treatment-related data, with results expressed as the mean ± standard deviation for normally distributed variables or the median with interquartile range for non-normally distributed variables. For comparisons between demographic and treatment variables, chi-square tests or Fisher’s exact tests were employed as appropriate. Paired *t*-tests and chi-square tests were utilized to assess differences between the groups before and after the interventional treatment. Additionally, univariate analysis and multivariate logistic regression models were constructed to determine factors contributing to the occurrence of N/V. Variables with *p*-values < 0.1 in the univariate analysis were included in the multivariate model. Multicollinearity was assessed using the variance inflation factor (VIF). A *p*-value of less than 0.05 was considered to indicate statistical significance.

## 3 Results

### 3.1 Prophylactic use of PH + DXMS reduced the incidence of post-intervention N/V in patients with HCC

The sociodemographic data of the patients are shown in Table 1. There were no significant differences between the PH + DXMS group and the PH group in terms of gender, age, body mass index (BMI), history of smoking, history of underlying diseases, Eastern Cooperative Oncology Group (ECOG) score, tumor-node-metastasis (TNM) stage, liver function, duration of hospitalization, white blood cells (WBC), red blood cells (RBC), hemoglobin (Hb), or platelets (PLT) (all *p* > 0.05).

**Table 1:** General information of patients.

Variables	Total (*n* = 112)	Control (*n* = 67)	PH + DXMS (*n* = 45)	*p*
Gender, male	94 (83.9%)	56 (83.6%)	38 (84.4%)	0.903
Age	61.63 ± 10.78	62.00 ± 10.80	61.07 ± 10.86	0.655
Body mass index (kg/m^2^)	22.63 (20.77, 25.08)	22.27 (20.07, 25.33)	23.23 (21.55, 24.96)	0.183
Body mass index (*n*%)				
<24	70 (62.5%)	43 (64.2%)	27 (60.0%)	0.654
≥24	42 (37.5%)	24 (35.8%)	18 (40.0%)	
Smoking history	56 (50%)	34 (50.7%)	22 (48.9%)	0.847
History of underlying disease	91 (81.3%)	52 (77.6%)	39 (86.7%)	0.229
Hepatitis B	72	41	31	
Cirrhosis	12	7	5	
Hypertension	36	21	15	
Gout	3	2	1	
Diabetes	23	18	5	
Other	14	10	4	
ECOG score				
0	108 (96.4%)	64 (95.5%)	44 (97.8%)	0.648
1	4 (3.6%)	3 (4.5%)	1 (2.2%)	
TNM stage				
Ia	4 (3.6%)	4 (6.0%)	0	0.052
Ib	5 (4.5%)	3 (4.5%)	2 (4.4%)	
IIa	15 (13.4%)	7 (10.4%)	8 (17.8%)	
IIb	16 (14.3%)	10 (14.9%)	6 (13.3%)	
IIIa	10 (8.9%)	2 (3.0%)	8 (17.8%)	
IIIb	61 (54.5%)	40 (59.7%)	21 (46.7%)	
IV	1 (0.9%)	1 (1.5%)	0	
LFC				
A	82 (73.2%)	50 (74.6%)	32 (71.1%)	0.798
B	29 (25.9%)	16 (23.9%)	13 (28.9%)	
C	1 (0.9%)	1 (1.5%)	0	
Days in hospital	6.12 ± 3.22	5.96 ± 2.85	6.36 ± 3.74	0.522
WBC	4.99 ± 2.46	5.28 ± 2.79	4.57 ± 1.83	0.135
RBC	4.16 ± 0.65	4.16 ± 0.69	4.16 ± 0.59	0.955
Hb	129.08 ± 19.99	128.71 ± 20.21	129.62 ± 19.87	0.815
PLT	125.00 (84.00, 169.00)	125.50 (87.00, 193.50)	125.00 (83.50, 155.50)	0.238

DXMS, dexamethasone; PH, palonosetron hydrochloride; WBC, white blood cell; RBC, red blood cell; Hb, hemoglobin; PLT, platelet; LFC, liver function classification. *p* < 0.05 was considered a statistically significant difference between groups.

The incidence of N/V in patients with HCC before and after TACE was analyzed. In the PH group, the incidence of nausea and vomiting (*p* < 0.001 and *p* = 0.003, respectively) increased significantly before and after TACE. Patients who received PH + DXMS experienced a significant reduction in the incidence of nausea post-TACE (67.2% vs. 37.8%, respectively; *p* = 0.004), and there was also an improvement in the frequency of vomiting (26.9% vs. 8.9%, respectively; *p* = 0.060) (Table 2).

**Table 2:** Efficacy of antiemetics on nausea and vomiting in HCC patients undergoing TACE.

Variables	Control (*n* = 67)			PH + DXMS (*n* = 45)		
	Pre-intervention	Post-intervention	*p*	Pre-intervention	Post-intervention	*p*
Nausea			<0.001			<0.001
0	61 (91.0%)	22 (32.8%)		45 (100.0%)	28 (62.2%)^**^	
1	5 (7.5%)	15 (22.4%)		0	7 (15.6%)	
2	1 (1.5%)	30 (44.8%)		0	9 (20.0%)	
3	0	0		0	1 (2.2%)	
Vomiting			0.003			0.117
0	64 (95.5%)	49 (73.1%)		45 (100.0%)	41 (91.1%)	
1	2 (3.0%)	5 (7.5%)		0	1 (2.2%)	
2	1 (1.5%)	13 (19.4%)		0	3 (6.7%)	

DXMS, dexamethasone; PH: palonosetron hydrochloride; TACE, transcatheter arterial chemoembolization. *p* < 0.05 was considered a statistically significant difference between groups. ^**^p < 0.01 compared with the control group.

### 3.2 Factors influencing nausea in patients with HCC

To identify the factors influencing the efficacy of PH + DXMS in alleviating TACE-induced nausea among HCC patients, all patients in the PH + DXMS group were categorized into a nausea group (*n* = 17) and a non-nausea group (*n* = 28), and univariate analysis were conducted. Baseline characteristics including gender, age, BMI, history of smoking, history of underlying diseases, ECOG score, TNM stage, liver function, WBC, RBC, Hb, or PLT counts were compared. Baseline analysis showed no significant differences between the nausea group and the non-nausea group (*p* > 0.05), except for the length of hospital stay (*p* = 0.031) (Table 3).

**Table 3:** Univariate analysis of the occurrence of nausea after TACE in the PH + DXMS group.

Variables	Non-nausea (*n* = 28)	Nausea (*n* = 17)	*p*
Gender, male	24 (85.7%)	14 (82.4%)	1.000
Age	62.00 (55.50, 68.00)	64.00 (48.00, 72.50)	0.782
Body mass index (kg/m^2^)	23.53 (22.03, 24.98)	22.04 (20.24, 24.88)	0.076
Body mass index (*n*%)			
<24	16 (57.1%)	11 (64.7%)	0.616
≥24	12 (42.9%)	6 (35.3%)	
Smoking history	16 (57.1%)	6 (35.3%)	0.155
History of underlying disease	26 (92.9%)	13 (76.5%)	0.179
Hepatitis B	21	10	
Cirrhosis	3	2	
Hypertension	11	4	
Gout	0	1	
Diabetes	4	1	
Other	4	0	
ECOG score			
0	28 (100.0%)	16 (94.1%)	0.378
1	0	1 (5.9%)	
TNM stage			
Ia	0	0	0.601
Ib	2 (7.1%)	0	
IIa	5 (17.9%)	3 (17.6%)	
IIb	4 (14.3%)	2 (11.8%)	
IIIa	3 (10.7%)	5 (29.4%)	
IIIb	14 (50.0%)	7 (41.2%)	
IV			
LFC			
A	22 (78.6%)	10 (58.8%)	0.188
B	6 (21.4%)	7 (41.2%)	
C	0	0	
Days in hospital	5.43 ± 2.61	7.88 ± 4.78	0.031
WBC	4.40 ± 2.01	4.85 ± 1.49	0.427
RBC	4.23 ± 0.64	4.03 ± 0.47	0.265
Hb	133.86 ± 19.65	122.65 ± 18.74	0.066
PLT	122.61 ± 52.93	133.24 ± 41.60	0.485
Pre-intervention ALT (U/L)	27.50 (20.25, 32.00)	38.00 (25.50, 47.00)	0.069
Post-intervention ALT (U/L)	138.38 ± 207.70	106.33 ± 119.46	0.595
Pre-intervention AST (U/L)	31.00 (27.25, 44.25)	44.00 (25.50, 58.50)	0.126
Post-intervention AST (U/L)	145.67 ± 247.77	159.93 ± 202.28	0.856

ALT, alanine aminotransferase; AST, aspartate aminotransferase; DXMS, dexamethasone; PH, palonosetron hydrochloride; WBC, white blood cell; RBC, red blood cell; Hb, hemoglobin; PLT, platelet; LFC, liver function classification; TACE, transcatheter arterial chemoembolization. *p* < 0.05 was considered a statistically significant difference between groups.

The univariate analysis yielded the following variables within the set threshold range (*p* < 0.1): length of hospital stays (*p* = 0.031), BMI (*p* = 0.076), Hb (*p* = 0.066), and alanine aminotransferase (ALT) pre-intervention (*p* = 0.069). These were incorporated into the multivariate logistic regression analysis, which revealed that ALT > 40 U/L pre-intervention was an independent influencing factor for nausea in patients with HCC (OR = 5.833, 95% CI: 1.252–27.170, *p* = 0.025).

## 4 Discussion

TACE has emerged as a prevalent method for managing HCC, and nausea and vomiting stand out among the most frequent and clinically significant adverse effects during such treatment. Recognizing this concern, various global guidelines focusing on N/V have been formulated (14, 15). These guidelines consistently recommend the use of antiemetic drugs such as 5-HT3RA, DXMS, aprepitant, and fosaprepitant, either as monotherapy when patients experience mild nausea or vomiting or as combination therapy in severe conditions. However, there is limited research on the actual implementation of these guidelines in clinical practice. Therefore, the objective of this study was to investigate the clinical use and effectiveness of the second-generation 5-HT3RA-PH in conjunction with DXMS following TACE in patients with HCC. An additional aim of the study was to identify factors influencing N/V in these patients.

The use of 5-HT3 receptor antagonists can be traced back to 1952, when the potential benefits of 5-HT via receptor interactions were first proposed (16). Gaddum and Picarelli (17) later identified the action of 5-HT on the m receptor in neural tissues, marking the discovery of the 5-HT3 receptor. This knowledge paved the way for the development of 5-HT3RA, with the selective antagonistic effect of the 5-HT3 receptor particularly noted for its relationship with cisplatin-induced vomiting inhibition, fostering the rapid development and expansion of this class of drugs (18). First-generation 5-HT3RAs demonstrated efficacy in controlling chemotherapy-induced N/V; however, their clinical utility was limited by their short half-life and high cost (19).

In contrast, PH, as a second-generation 5-HT3RA, offered unique advantages as it was shown to have better physical and chemical stability during Y-site administration (20, 21) and improved efficacy in preventing early and late postoperative nausea and vomiting when compared to first-generation 5-HT3RAs like ondansetron (22). Additionally, PH has proven to be cost-effective for sustained usage over a period of 3 to 4 days, offering better suppression of nausea (23). Moreover, PH significantly reduces the incidence of nausea and vomiting in patients post-TACE, with fewer adverse reactions and high safety (24). It has been reported that the risk of drug interactions with PH is minimal, and it can be safely combined with corticosteroids, analgesics, and other medications. No significant pharmacokinetic interactions were observed when PH was co-administered with DXMS, aprepitant, or metoclopramide (25).

An anabolic steroid known for its anti-inflammatory, anti-allergic, and immunosuppressive properties, DXMS has long been used to prevent and treat chemotherapy-induced N/V (26). The use of DXMS in doses as low as 4 to 5 mg for effective N/V reduction has been substantiated by clinical evidence. Moreover, its prolonged action renders it a viable alternative for managing late-onset symptoms (27). DXMS, due to its immunosuppressive effects, may induce or exacerbate infections, particularly in cancer patients with compromised immune function, thereby increasing the risk of infection. However, existing studies indicate that the use of DXMS during TACE does not increase the incidence of post-TACE infections, making its use in TACE treatment safe (28). Some studies report that both monotherapy with PH and combined PH with DXMS are equally effective in preventing postoperative N/V following laparoscopic surgery, whereas DXMS alone demonstrates the poorest effect (29). However, these findings have not been confirmed in patients with HCC. Moreover, the combination of DXMS and PH has been shown to be more effective than PH alone in preventing N/V, and patients receiving this combination therapy require fewer postoperative antiemetic medications (30, 31).

Our findings corroborate existing literature, demonstrating that the prophylactic use of PH in combination with DXMS significantly reduces the incidence of TACE-induced nausea when compared to the use of PH alone in patients with HCC. These results are consistent with the findings reported by Lu et al. (28) and Sakamoto et al. (32), reinforcing the clinical value of this combination therapy in enhancing the quality of life of the patient during treatment. TACE serves as a palliative treatment for advanced primary and metastatic liver cancer. However, the nausea and vomiting induced by interventional therapy can adversely affect the quality of life for patients undergoing this treatment (33–35). The combination of PH and DXMS not only decreases the incidence of nausea but also reduces the requirement for rescue antiemetics and contributes to shorter hospitalization, thereby improving overall patient tolerance to TACE and facilitating treatment adherence. Although the prophylactic use of PH + DXMS is established, our findings provide practical guidance for HCC patients undergoing TACE and identify ALT as a potential biomarker for risk stratification, enhancing individualized patient care.

This study identified elevated pre-TACE ALT levels (>40 U/L) as an independent factor influencing the occurrence of N/V. This finding underscores the significance of considering liver function as a critical parameter in the management of these side effects. Elevated ALT, indicative of hepatocellular injury and inflammation (36), reflects underlying hepatic impairment that may also disrupt the metabolism and clearance of chemotherapeutic, embolic, and antiemetic agents such as PH and DXMS, resulting in variability in treatment response. Notably, the local vascular microenvironment during TACE plays a crucial role in determining the pharmacokinetics of both chemotherapeutic and antiemetic drugs. As highlighted by Lungu et al. (37) factors such as diffusion rate, lipophilicity, and clearance kinetics significantly influence local drug delivery and systemic exposure. Hepatic arterial embolization can alter liver perfusion and reduce first-pass metabolism, thereby affecting the pharmacokinetic behavior of antiemetic agents. PH, characterized by high receptor affinity and a prolonged plasma half-life, (38) may maintain its efficacy even under altered hepatic blood flow, whereas DXMS may exhibit prolonged systemic exposure in patients with hepatic impairment or elevated ALT. These pharmacokinetic differences underscore the importance of considering hepatic physiology when optimizing antiemetic regimens in interventional oncology. These findings collectively suggest that elevated ALT reflects both hepatic injury and altered pharmacodynamics, emphasizing the need for careful monitoring and management of HCC patients with impaired liver function, particularly since interventional therapies can further exacerbate hepatic stress (39, 40). Despite the encouraging results, the potential risks of adverse reactions associated with PH and DXMS highlight the necessity for continuous clinical vigilance and further research to refine and optimize antiemetic protocols for patients undergoing treatment for HCC.

### 4.1 Limitations of the study

Although our study provides valuable clinical evidence for the combined use of PH and DXMS, it has several limitations. First, this study was a retrospective analysis conducted at a single institution, which inevitably introduces potential selection bias and limits the external validity of the findings. Second, the relatively small sample size and the male-dominated population may restrict the generalizability of the results. For instance, although the difference in vomiting incidence between the PH and PH + DXMS groups did not reach statistical significance (*p* = 0.06), the downward trend suggests a potential clinical relevance. The limited sample size may have restricted statistical power, and it is plausible that this effect would achieve significance in a larger, prospective cohort. Future research should include larger, multicenter, prospective trials with a more balanced gender distribution to provide stronger evidence and broader applicability. Moreover, only two regimens, PH alone and PH combined with DXMS, were evaluated. Other potential antiemetic protocols, such as DXMS monotherapy or the addition of NK1 receptor antagonists, were not included. Future studies should compare multiple regimens to determine the most effective and cost-efficient antiemetic strategy for patients undergoing TACE. Additionally, further mechanistic studies are crucial to elucidate the biochemical pathways involved in the combined therapy, particularly in terms of how this regimen modulates neuroendocrine pathways related to nausea and vomiting. For instance, whether the combination improves symptoms by inhibiting specific neurotransmitters or activating relevant receptors warrants further exploration. Future research should also assess the impact of PH + DXMS combination therapy on other quality-of-life indicators, such as appetite and fatigue. Moreover, while DXMS is generally considered safe, detailed analyses of potential adverse effects such as infection risk or metabolic disturbances were beyond the scope of this study. Future studies should include multidimensional outcome assessments and comprehensive safety monitoring to better capture the clinical impact of antiemetic therapy. Finally, future investigations could explore the potential of ALT as a biomarker for predicting nausea and vomiting.

## 5 Conclusion

In conclusion, despite the prophylactic use of PH in patients with HCC, the incidence of post-intervention N/V remained relatively high. The combined application of PH and DXMS was more effective in improving the rate of occurrence of N/V. Pre-TACE ALT levels > 40 U/L may be an independent risk factor for nausea in patients with HCC, underscoring the need for clinical healthcare workers to be aware of this factor in the clinical management of such patients. Given the retrospective design and relatively small, male-dominated sample, these findings should be interpreted cautiously. Future studies should focus on larger, multicenter, prospective trials to validate these risk factors, explore additional predictive biomarkers, and investigate optimized dosing regimens or alternative antiemetic agents to further improve patient outcomes.

## Data Availability

The raw data supporting the conclusions of this article will be made available by the authors, without undue reservation.
